# The Consequence of Combined Pain and Stress on Work Ability in Female Laboratory Technicians: A Cross-Sectional Study

**DOI:** 10.3390/ijerph121215024

**Published:** 2015-12-11

**Authors:** Kenneth Jay, Maria Kristine Friborg, Gisela Sjøgaard, Markus Due Jakobsen, Emil Sundstrup, Mikkel Brandt, Lars Louis Andersen

**Affiliations:** 1 Department of Physical Activity and Health, Institute for Sports Science and Clinical Biomechanics, University of Southern Denmark, Odense 5230, Denmark; gsjogaard@health.sdu.dk; 2 National Research Centre for the Working Environment, Copenhagen 2100, Denmark; mkf@nrcwe.dk (M.K.F.); mdj@nrcwe.dk (M.D.J.); esu@nrcwe.dk (E.S.); mbp@nrcwe.dk (M.B.); lla@nrcwe.dk (L.L.A.); 3 Physical Activity and Human Performance group, SMI Department of Health Science and Technology, Aalborg University, Aalborg 9220, Denmark

**Keywords:** pain stress relationship, behavior, social factors, fear-avoidance, biopsychosocial, learned helplessness, resources and demands

## Abstract

Musculoskeletal pain and stress-related disorders are leading causes of impaired work ability, sickness absences and disability pensions. However, knowledge about the combined detrimental effect of pain and stress on work ability is lacking. This study investigates the association between pain in the neck-shoulders, perceived stress, and work ability. In a cross-sectional survey at a large pharmaceutical company in Denmark 473 female laboratory technicians replied to questions about stress (Perceived Stress Scale), musculoskeletal pain intensity (scale 0–10) of the neck and shoulders, and work ability (Work Ability Index). General linear models tested the association between variables. In the multi-adjusted model, stress (*p* < 0.001) and pain (*p* < 0.001) had independent main effects on the work ability index score, and there was no significant stress by pain interaction (*p* = 0.32). Work ability decreased gradually with both increased stress and pain. Workers with low stress and low pain had the highest Work Ability Index score (44.6 (95% CI 43.9–45.3)) and workers with high stress and high pain had the lowest score (32.7 (95% CI 30.6–34.9)). This cross-sectional study indicates that increased stress and musculoskeletal pain are independently associated with lower work ability in female laboratory technicians.

## 1. Introduction

In the working population reduced work ability caused by physical or mental disabilities is highly prevalent and is the leading cause of long-term sick leave [1,2,3] staff turnover and early retirement [4,5]. Reduced work ability is not only costly for the individual, but also for the organization and society due to lost productivity and compensation costs [3]. Work ability is the result of the interaction between the employee and his or her work. Consequently, work ability can be described as the balance between the employee’s resources and the work demands [6]. The employee contributes to his or her work ability via health and functional abilities, knowledge, skills, attitude and motivation. The workplace influences work ability through organizational factors–through leadership, management issues, work demands and social factors [7]. On the basis of comprehensive clinical assessments and statistical analyses, Tuomi and colleagues identified a short set of questions ultimately resulting in a score indicating the employee’s work ability, aptly named the Work Ability Index (WAI) [8]. Ilmarinen and Tuomi have shown that people with high WAI scores have a lower risk for early retirement and a higher quality of life—even after retirement [6,7,9]. Furthermore, studies using the WAI have also presented data suggesting that it is possible to sustainably improve work ability—even at older age—provided the right measures are taken [6,7,9]. The WAI has been associated with musculoskeletal pain, chronic disease, work productivity, sickness, untimely retirement, as well as all-cause mortality [6,8]. In occupational groups where employees are exposed to repetitive, monotonous and/or forceful exertion, compromised body positions and/or insufficient recovery, there is an elevated risk of both impaired work ability and chronic musculoskeletal disorders in the long term [10]. 

Research on the relationship between stress and musculoskeletal pain has previously largely focused on back pain and multisite pain but only to a limited extent on neck-shoulder pain specifically. Oberlinner *et al.* demonstrated, by conducting a comprehensive survey combining questionnaire data and medical examinations in one division of a major chemical company in Germany, that although occupational stressors were perceived differently, there was no difference in the prevalence of back pain between different working groups within the company [11]. Conversely, a 2000 review by Davis and Haeney [12] on the relationship between psychosocial work characteristics and low back pain established that while it is problematic to argue for causal inferences, it appears that psychosocial characteristics are related to at least some lower back pain outcomes. Furthermore, employees’ reactions to psychosocial work characteristics (e.g., job dissatisfaction and job stress) are more consistently related to lower back pain than the psychosocial work characteristics themselves. In conjunction with a systematic review by Hoogendroom *et al*. on the psychosocial factors at work and in private life as risk factors for back pain, there is confirmation for an effect of work-related psychosocial factors on the perception of physical well-being [13]. Congruent with this, Lindegaard *et al.* reported a combination of frequent pain and stress constituted the highest risk for a decrease in work performance evaluated by a single item from the WAI questionnaire and conclude that workplace interventions should focus on promoting musculoskeletal well-being and encompass both individual and organizational interventions to minimize the risk of increased work-related stress [14]. Furthermore, Ganster *et al.* [15], and Cohen *et al.* [16,17] concluded that there are three main approaches to the definition and study of work stress. In brief, the first approach conceptualizes stress as a characteristic of the environment that affects the individual. The second approach defines stress as the psychological reaction (psychological, physiological and behavioral) on environmental stimuli. The third and most prevalent approach views stress as the interaction between environmental characteristics and the subjective reaction to these characteristics [15]. In the present study, we used the third definition as we defined psychological stress as a process by which environmental stimuli initiate cognitive and physiological reactions that ultimately can affect wellbeing. Psychological stress occurs when an individual perceives that environmental demands exceed his or her adaptive capacity [16]. The aim of the present study was to investigate the association between pain in the neck and shoulders, stress and work ability measured by the WAI. 

## 2. Experimental Section 

### 2.1. Study Design

This study is an explorative analysis of baseline data obtained during a worksite intervention trial previously described by our research team [18]. Data for this study were collected during the spring of 2014.

### 2.2. Ethics

Ethical approval was obtained from The Danish National Committee on Biomedical Research Ethics (The local ethical committee of Frederiksberg and Copenhagen; H-3-2010-062) as part of the research program “Implementation of physical exercise at the workplace (IRMA)”. The trial “Implementation of physical exercise at the Workplace (IRMA09)—Laboratory technicians” was registered in the ClinicalTrials.gov register (NCT02047669) prior to participant enrolment. All experimental conditions conformed to The Declaration of Helsinki.

### 2.3. Participants

Out of 756 laboratory technicians at a large pharmaceutical company in Denmark, 539 completed questionnaires on musculoskeletal pain, perceived level of stress and work ability. Of these, 473 were women and included in the analysis. Table 1 shows participant demographics of relevant data. All eligible participants were informed about the purpose and content of the study. Table 1 shows participant characteristics of relevant data.

**Table 1:** Descriptive characteristics of the female laboratory technicians included in the analysis.

Descriptive Characteristics	Mean (S.D.)
Number of included observations	473
Age, years	46.0 (9.5)
Body mass index(kg·m^−2^)	24.2 (3.7)
Smokers, percentage	1.9 (0.3)
Seniority, years	20.9 (10.8)
Number of working hours per week	36.9 (4.3)
Neck-shoulder pain intensity (0–10 VAS-scale)	2.6 (2.4)
The Perceived Stress Scale score (0–40)	12.3 (6.4)
Work Ability Index score (7–49)	41.8 (4.9)

### 2.4. Work Ability

The WAI consists of seven indicators providing a score between 7 and 49 (higher is better) that gauge occupational well-being by providing subjective estimations on work ability. This includes capability of employees to perform their work tasks with respect to demands, health, as well as mental resources [6,19]. Further, the WAI includes the dimensions of the determinants (e.g., health) and outcomes (sick leave and functional limitations) of work ability. Currently, the WAI has been translated into 26 languages and is used in numerous countries throughout the world [20]. The validity of WAI is well documented [21] and the test-retest reliability have been found to be a stable measure [22]. For reference, we have classified the sum score into the following three categories of work ability: Poor (score 7–27), Moderate (score 28–36), Good/Excellent (score 36–49). 

### 2.5. Pain

We asked the participants to rate pain intensity in the neck and shoulder on a modified VAS scale (0–10). For reference, “0” is “no pain” and “10” is “worst imaginable pain”. The neck and shoulder were defined by drawings from the Nordic Questionnaire [23] and an average pain score of the two regions was subsequently calculated and used in the statistical analysis. We defined the following cut points for the level of pain: (0 < low pain ≤ 2), (2 < moderate pain ≤ 5), and (high pain > 5) [24,25,26,27].

### 2.6. Stress

The Perceived Stress Scale (PSS), developed by Cohen, Kamarck, and Mermelstein is a comprehensive stress questionnaire and was designed to measure “the degree to which individuals appraise situations in their lives as stressful”. Items evaluate the degree to which people find that life is unpredictable, uncontrollable, or overloaded [28]. These three aspects have been confirmed as vital elements of the experience of stress and provide a thorough insight into the degree of learned helplessness experienced by the individual [29,30,31,32,33]. The Perceived Stress Scale includes questions intended to evaluate the current level of stress experienced by the subject. The PSS-10 is an abbreviated version of the scale, consisting of only 10 items (the full version has 14 items), administered in only a few minutes, and easily scored. Because the perceived stress scale assess general beliefs about perceived stress without providing subjects with a list of specific life events, scores are not biased by event content or by differential recall of previous life experiences. In brief, each item on the PSS-10 questionnaire is rated on a 5-point Likert scale ranging from “never” (0) to “almost always” (4). Positively worded items are reverse scored, and the ratings are summed, with higher scores indicating more perceived stress. The PSS-10 score is obtained by reversing the scores on the four positive items: For example, 0 = 4, 1 = 3, 2 = 2, *etc.* and then summing across all 10 items. A score of 13 is considered average and stress scores of more than 20 indicate high stress [28]. For reference, we divided the scoring into three categories with the following cut-off points: (low stress ≤ 10), (10 < moderate stress ≤ 20) and (high stress > 20). Examples of questions from the PSS-10 questionnaire include: (i) “In the past month, how often have you been angry because of things that happened that were outside of your control?”, (ii) “In the past month, how often have you felt that things were going your way?” and (iii) In the past month, how often have you felt unable to control the important things in your life?” [28].

### 2.7. Statistics

We performed all statistical analyses using the SAS statistical software for Windows (SAS Institute, Cary, NC, USA). Using the General Linear Modelling procedure (proc GLM), we analyzed the following relationships between stress, pain and WAI. (1) Stress to pain with neck-shoulder pain as the dependent variable; (2) stress to WAI with WAI as the dependent variable; (3) pain to WAI with WAI as the dependent variable and (4) stress + pain, and stress by pain interaction to WAI with WAI as the dependent variable. All analyses were controlled for age. To assess simple associations, we also used the CORR procedure to determine Pearson’s correlation coefficients. An alpha level of <0.05 was accepted as statistically significant. Results are reported as least square means (95% confidence limits).

## 3. Results

**Figure 1:**
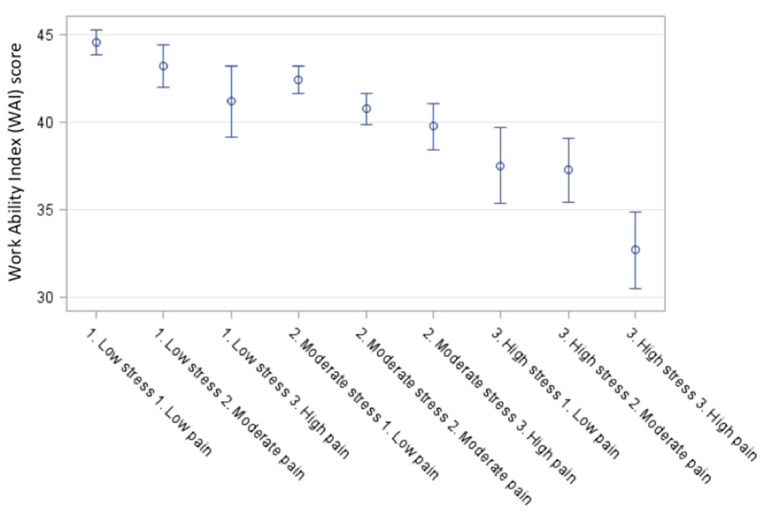
Least square means stress + pain plot with WAI as the dependent outcome variable. As stress and neck-shoulder pain increase, the work ability of female laboratory technicians decreases. Developing chronic musculoskeletal pain while experiencing high stress returns the lowest work ability score.

Stress and neck-shoulder pain were positively correlated with a Pearsons correlation coefficient of 0.29 (*p* < 0.0001). Additionally, stress was independently correlated to WAI with a coefficient of −0.51 (*p* < 0.0001) indicating a lower WAI score with increased stress. Similarly, chronic neck-shoulder pain also correlated to WAI with a coefficient of −0.36 (*p* < 0.0001) indicating a lower WAI score with increased neck-shoulder pain. Figure 1 shows a stress + pain plot with WAI as the dependent outcome variable. 

In the multi-adjusted model, both stress (*p* < 0.001) and pain (*p* < 0.001) had independent main effects on WAI, and there was no significant stress by pain interaction (*p* = 0.32). WAI decreased gradually with both increased stress and pain. Workers with low stress and low pain (n = 133) had the highest work ability (44.6 (95% CI 43.9–45.3)) and workers with high stress and high pain (n = 14) had the lowest (32.7 (95% CI 30.6–34.9)). WAI is therefore dependent on the magnitude of both stress and neck-shoulder pain in an additive fashion. In this population, WAI score decreased 26.6% from low stress + low pain to high stress + high pain.

## 4. Discussion

Our study showed that increased stress and musculoskeletal pain were independently associated with lower work ability in female laboratory technicians. 

### 4.1. Stress and Pain

Stress and neck-shoulder pain were associated with work ability in an additive fashion. This result supports that psychosocial factors interact with somatic problems and is congruent with studies reporting a correlation between back pain and psychological stress [11,12,34,35]. As most research on stress and musculoskeletal pain has focused on back pain and multisite pain, this study contributes to the field by indicating an association specifically between neck-shoulder pain and perceived stress. Due to the cross-sectional nature of the present study it is not possible to infer about causality between stress and neck-shoulder pain, but research suggests that stress and pain are positively associated [11]. Workers with neck-shoulder pain may be more likely to perceive themselves as more stressed, as pain potentially limits normal social interactions and work ability [36]. Possibly, perceived stress intensifies or causes neck-shoulder pain through the physical response when subjected to psychological stress. Psychological stress activates the sympathetic nervous system, which releases noradrenaline from widely distributed synapses and adrenaline from the adrenal medulla. The secretion of these hormones primes the body for action causing the muscles tense up, blood vessels to constrict and blood pressure to increase [37,38,39,40,41].

### 4.2. Stress, Pain and Work Ability

To our knowledge, only a few studies have examined the interaction between musculoskeletal pain and perceived stress on work ability [11,14] making the present study the first study to show the combined effect of neck-shoulder pain specifically, and perceived stress on work ability. Our study showed that stress and pain did not interact in relation to work ability, but that the effect was additive. The result implicate that an intervention strategy aimed at reducing both neck-shoulder pain and perceived stress may be beneficial to prevent reduced work ability and is congruent with other studies attenuating multifactorial intervention strategies [42,43]. Several randomized controlled trials have reported positive reductions in chronic pain in the upper extremity following both strenuous and non-strenuous resistance training using both weights and elastic bands [44,45,46,47,48,49,50], thus providing insight into factors positively affecting work ability and factors reducing both chronic and non-chronic musculoskeletal pain. For instance, in a recent wide-ranging study Kettunen *et al.* showed that low- to moderate physical activity coupled with improvements in cardiorespiratory fitness, are closely associated with long-term (12 months) improved work ability in subjects with low-, moderate- and good work ability at baseline [7]. The researchers measured cardiorespiratory fitness and WAI at baseline, 4 months, 8 months and 12 months in 338 participants and found 2%–3% increases in WAI, at 4 months, again at 8 months and again at 12 months in the exercise training group, whereas the control group experienced a 2% decrease in WAI score [7]. Although the study by Kettunen and colleagues did not include participants with chronic pain, it shows that physical fitness interventions can have a positive effect on work ability. However, as work ability appears to be comprised of a complex set of interactions between psychological, environmental, social and biological factors, single-component interventions are limited as they do not target all aspects of the biopsychosocial model in relation to work ability. Arguably, interventions aimed at promoting work ability do not only have to be multifaceted but must also be implemented on an organizational level [51,52]. Psychological or physiotherapeutic treatment outside of work or in work hours with no involvement of the organization may not be ideal for improving work ability. Involvement of the organization may imply social and emotional support in conjunction with acceptance from the organizational leader. Furthermore, organizational involvement can include a restructuring of work factors that contribute to the employee’s pain and stress. As both psychological stress and neck-shoulder pain are highly prevalent [53,54], future research should investigate the effect of multifaceted interventions implemented on an organizational level and targeted specifically at neck-shoulder pain and stress to promote work ability.

### 4.3. Limitations

This study demonstrates the combined effect of neck-shoulder pain specifically, and perceived stress on work ability. However, some limitations exist. First, the cross-sectional design does not permit examination of causal relationships. Thus, prospective studies should assess the combined effect of stress and pain on deterioration of work ability. On the other hand, high stress and pain may reflect that the worker has already experienced deterioration of work ability. Nevertheless, the results are still of interest, as they give an insight into factors associated with work ability, which can be used in the development of preventive intervention strategies. Second, sample size is relatively small limiting statistical power and the reliability of results. Third, self-reported data are a limitation as they may be influenced by subjective factors. Fourth, given the demographic characteristics of this sample (Danish female laboratory technicians) generalizability remains to be determined. The presented results may not be generalizable to male employees, as gender may modify the examined relations. Conversely, using a homogenous sample consisting of female laboratory technicians is also a noteworthy strength as it limits bias from socioeconomic confounding.

## 5. Conclusions 

This cross-sectional study confirms a necessity to look at both stress and musculoskeletal pain as combined co-factors when implementing interventional strategies to promote work ability.
